# ^1^H NMR-Based Analysis of Serum Metabolites in Monocrotaline-Induced Pulmonary Arterial Hypertensive Rats

**DOI:** 10.1155/2016/5803031

**Published:** 2016-01-18

**Authors:** Taijie Lin, Jinping Gu, Caihua Huang, Suli Zheng, Xu Lin, Liangdi Xie, Donghai Lin

**Affiliations:** ^1^Fujian Hypertension Research Institute, First Affiliated Hospital of Fujian Medical University, Fuzhou 350005, China; ^2^MOE Key Laboratory of Spectrochemical Analysis & Instrumentation, Key Laboratory for Chemical Biology of Fujian Province, College of Chemistry and Chemical Engineering, Xiamen University, Xiamen 361005, China; ^3^The Physical Education Department, Xiamen University of Technology, Xiamen 361024, China

## Abstract

*Aims*. To study the changes of the metabolic profile during the pathogenesis in monocrotaline (MCT) induced pulmonary arterial hypertension (PAH).* Methods*. Forty male Sprague-Dawley (SD) rats were randomly divided into 5 groups (*n* = 8, each). PAH rats were induced by a single dose intraperitoneal injection of 60 mg/kg MCT, while 8 rats given intraperitoneal injection of 1 ml normal saline and scarified in the same day (W0) served as control. Mean pulmonary arterial pressure (mPAP) was measured through catherization. The degree of right ventricular hypertrophy and pulmonary hyperplasia were determined at the end of first to fourth weeks; nuclear magnetic resonance (NMR) spectra of sera were then acquired for the analysis of metabolites. Principal component analysis (PCA) and orthogonal partial least-squares discriminant analysis (OPLS-DA) were used to discriminate different metabolic profiles.* Results*. The prominent changes of metabolic profiles were seen during these four weeks. Twenty specific metabolites were identified, which were mainly involved in lipid metabolism, glycolysis, energy metabolism, ketogenesis, and methionine metabolism. Profiles of correlation between these metabolites in each stage changed markedly, especially in the fourth week. Highly activated methionine and betaine metabolism pathways were selected by the pathway enrichment analysis.* Conclusions*. Metabolic dysfunction is involved in the development and progression of PAH.

## 1. Introduction 

Pulmonary arterial hypertension (PAH), a chronic and fatal disease with a poor prognosis, is characterized by elevated pulmonary arterial pressure which could lead to right side heart failure [1–3]. The pathophysiological changes of PAH, including migration and proliferation of pulmonary arterial smooth muscle cell (PASMC), adventitial fibroblast (PAAF), and leukocytes infiltration as well as release of inflammatory cytokines, could result in pulmonary endothelial dysfunction and vascular remodeling in the small pulmonary arteries [2, 4]. However, the cause and mechanism of these proliferations and antiapoptosis in PAH is unknown. It was suggested that metabolic dysfunction is implicated in the pathogenesis of PAH [5, 6] and drug metabolism such as protandim, which can upregulate the expression of antioxidant enzymes, may be beneficial for PAH [6].

Recently, metabonomic technique has been applied to clinical practice including diagnosis, evaluation of severity, progression and prognosis of diseases, and estimation of efficacy of surgical and pharmacological treatment [7–9]. Metabonomic profile provides potential biomarkers for screening cardiovascular diseases, enhances accuracy of diagnosis of endometriosis, hyperlipidemia, and atherosclerosis [10–12]. In one previous study, ^1^H NMR-based metabonomic analysis was used to determine serum metabolic profiles in patients with hypertension and to assess relationships between serum lipoprotein particle compositions and blood pressure [12].

When peripheral blood returns to the heart and then passes through the lungs, it contains almost the same metabolites as in both organs. Metabonomic analyses of circulating plasma may, to some degree, reflect the metabolic profiles indicating pulmonary arterial disease. In our previous studies, we had established a rat PAH model by intraperitoneal injection of monocrotaline (MCT) [13–15]. In the present study, we performed NMR-based metabonomic analysis of sera to determine the shift of metabolic profiles during the occurrence of PAH and explore important metabolic pathways correlating with pulmonary hypertension.

## 2. Materials and Methods

### 2.1. Establishment of Rat PAH Model

PAH rat was induced by a single intraperitoneal injection of MCT in our laboratory as described previously according to Xie and colleagues [13–15], which is the most commonly used animal model in PAH studies [16]. The use and care of all animals were approved by the Animal Care and Use Committee and conformed to the Guiding Principles for the Use and Care of Laboratory Animals of the National Institute of Health. Forty male Sprague-Dawley (SD) rats weighing 200–230 g, obtained from Shanghai SLAC Laboratory Animal Ltd. Corp. (Permission #SCXK (Shanghai) 2003-0003, Shanghai, China), were randomly divided into 5 groups (eight rats in each group). All rats were kept at 22 ± 2°C and 55% ± 5% of humidified room with 12 h light-dark cycle. Eight rats given the same volume of intraperitoneal injection of 1 mL normal saline and sacrificed in the same day (W0) served as control group. The other rats were peritoneally injected with a low dose of MCT (60 mg/Kg; Sigma Chemicals, St. Louis, MO). After the injection, the rats were sacrificed weekly in order during four weeks of follow-up (W1, W2, W3, and W4), eight rats in each subgroup. Blood samples were collected and centrifuged at 3200 rcf for 5 min at room temperature. Then the sera were removed and stored at −80°C for the metabolites analyses.

The mean pulmonary artery pressure (mPAP) and right ventricle hypertrophy index (RVHI) of all rats were measured weekly as described previously [14, 15]. Arterial wall thickness (WT), wall diameter (WD), total vessel area (TVA), and vessel luminal area (VLA) were determined by the IPP6.0 software. Percentages of the WT and wall area WA were calculated by using the formulas of WT% = 2 × WT/WD × 100% and WA% = (TVA − VLA)/TVA × 100%.

### 2.2. ^1^H NMR Spectroscopy of Sera

The serum samples (300 *μ*L each) were mixed with 250 *μ*L of phosphate buffered saline (PBS; pH 7.4) and centrifuged at 132000 rcf for 10 min at 4°C. Approximately 500 *μ*L of supernatant was collected and then transferred into 5 mm NMR tube. All NMR spectra were recorded at 25°C on a Bruker Avance III 600 MHz NMR Spectrometer (Bruker, Swiss). Standard one-dimensional (1D) spectra were acquired using a single 90° pulse sequence with an irradiation on the water resonance during the relaxation delay (RD) of 4 s. The 90° pulse length was adjusted to about 10 *μ*s. Meanwhile, transverse relaxation-edited spectra of serum samples were acquired using the water-suppressed Carr-Purcell-Meiboom-Gill (CPMG) pulse sequence [RD-90°-(*τ*-180°-*τ*)_*n*_-ACQ] with water suppression. A fixed total spin-spin relaxation delay of 120 ms was used to attenuate broad NMR signals of slowly tumbling molecules with short *T*_2_ relaxation times and to retain signals of low molecular weight compounds. The spectral width was 12 KHz with an acquisition time per scan of 1.63 s, and a total of 256 transients were collected into 32 K data points for each spectrum [17, 18].

### 2.3. Data Processing and Multivariate Statistical Analysis

To exploit the metabolic information embedded in the spectra, each free induction decay (FID) was zero-filled to 64 k points and all ^1^H NMR spectra were multiplied by a 0.3 Hz exponential line-broadening function prior to Fourier transform according to the method previously described [12, 19]. The acquired spectra were manually phased, corrected for baseline distortion, and carefully aligned. The chemical shifts were referenced to the methyl group of lactate (CH3 *δ* = 1.33 ppm). All the 1D ^1^H NMR spectra were carefully aligned by the MestReNova software (Version 6.5, Mestrelab Research S. L., Spain). The spectral region of *δ* 9.0–0.6 was segmented into 2800 bins with a width of 0.003 ppm. The integrals from the region of *δ* 6.0–4.7 ppm were excluded to eliminate the effects of imperfect water suppression in all spectra. The integrals were normalized to the total integral of the spectrum.

The resulting bucketed data matrices were imported into SIMCA-P^+^ 12.0 software package (Umetrics AB, Sweden) for chemometric analysis. Pareto scaling was used to increase the importance of low-concentration metabolites without significant amplification of noise. Principle components analysis (PCA) was performed for identifying differences among the metabolic profiles of all samples. The first three principle components (PCs) were used to generate a score plot displaying the correlation matrices. Orthogonal signal correction partial least-squares discriminant analysis (OPLS-DA) was applied for one-to-one classification between any two groups using MATLAB (Version MATLAB 2011b, MathWorks, USA). The reliability of the OPLS-DA model was tested as described previously [20]. Two criteria were used to identify the specific metabolites. One was the variable importance in projection (VIP) of the OPLS-DA model, and the other one was the correlation coefficient (*r*) of the variable relative to the predictive component (*t*[1]) in the OPLS-DA model [17, 18, 20].

### 2.4. Relative Quantification and Statistical Analysis

For relative quantification, the intensities of each metabolite were calculated by using the relative integrals of each NMR spectrum and were represented as mean ± standard deviation. Distributions of the metabolite values were tested. The Student's *t*-test was used to examine the difference between two groups. Pearson's correlation between metabolites was analyzed in different stages, respectively. The SPSS18.0 software was used to perform statistical analysis (Chicago, IL, USA), and *P* < 0.05 was considered statistically significant. The pathway enrichment analysis was performed by a free and web-based tool MetaboAnalyst (http://www.metaboanalyst.ca/), which was based on the concentrations of the characteristic metabolites.

## 3. Results

### 3.1. Changes of mPAP, RVHI, WT%, and WA% after MCT Treatment

**Figure 1:**
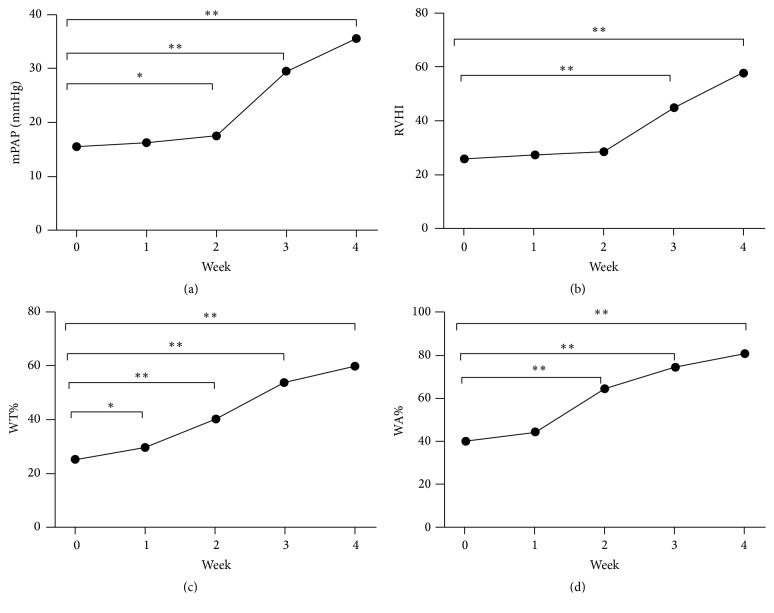
Changes in mPAP (a), RVHI (b), WT% (c), and WA% (d) in monocrotaline-induced rat PAH model. mPAP: mean pulmonary artery pressure; RVHI: right ventricle hypertrophy index; WT%: the ratio of vessel wall thickness and wall diameter; WA%: the ratio of vessel wall area and total vessel area. ^*∗*^ *P* < 0.05, ^*∗∗*^ *P* < 0.01.

At the end of the first week, WT% but not mPAP, RVHI, and WA% in rats was significantly altered compared to the W0 rats (*P* < 0.05). At the end of week 2, WT%, mPAP, and WA% increased remarkably, and these changes kept at higher level till the fourth week; all of the observed parameters were shown in Figures 1(a)–1(d). Elevation of pulmonary artery pressures, right ventricular hypertrophy, and pulmonary hyperplasia were prominent between 2 and 3 weeks after the MCT injection and were much higher in the 4th week, indicating that the experimental rats in weeks 2-3 were in the early stage of PAH, and PAH developed in the 4th week.

### 3.2. Changes in the Metabolic Profile in PAH Rats

**Figure 2:**
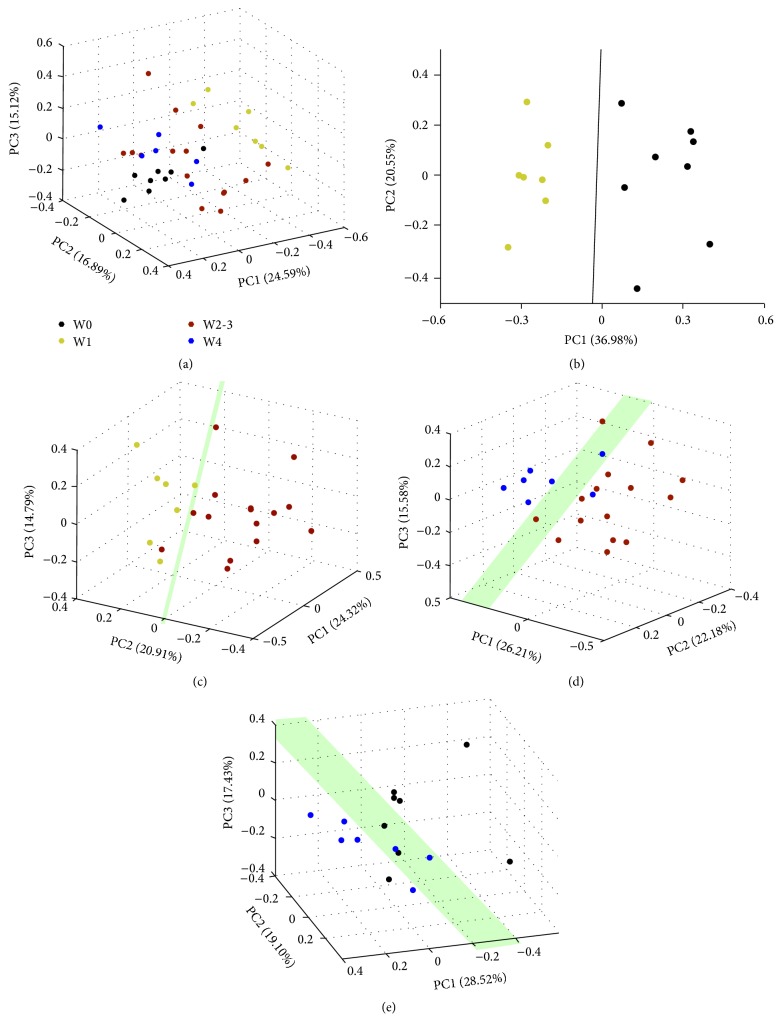
PCA score plots of ^1^H NMR data from serum of PAH rats. 3D PCA score plot generated from unsupervised PCA of NMR spectra of aqueous metabolites showing separate grouping for samples. Each point in the PCA score plot represents a specific individual sample, and samples with similar metabolic profiles are grouped together in clusters. (a) All rats; (b) W0 versus W1; (c) W1 versus W2-3; (d) W2-3 versus W4; (e) W4 versus W0.

**Figure 3:**
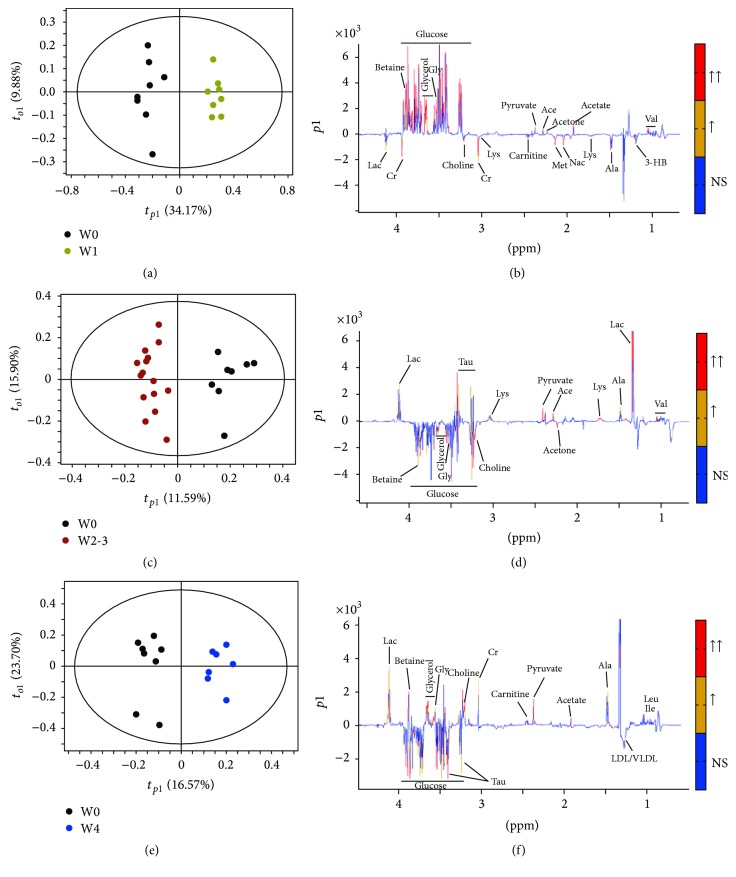
OPLS-DA score plots ((a), (c), and (e)) and OPLS-DA coefficients-coded loading plots ((b), (d), and (f)). Colors on the loading plots are used to identify the altered metabolites between two groups. When the |*r*| is greater than the critical value of *P* = 0.01 and the VIP > 1, the peaks were marked with red color; when the |*r*| is between the critical values of *P* = 0.01 and *P* = 0.05, and the VIP > 1, the peaks were marked with orange color; when the |*r*| is less than the critical value of *P* = 0.05 or VIP < 1, the peaks were marked with blue one. (a) and (b) W1 versus W0; (c) and (d) W2-3 versus W0; and (e) and (f) W4 versus W0.

An exploratory PCA has been applied to obtain a comprehensive comparison of metabolic profiles of the samples (Figure 2(a)). The PCA scores plot showed a differential tendency of metabolic profiles among groups in the course of the PAH process in rats. Because there was no difference in the metabolic profile between week 2 and week 3, we merged the data from week 2 and week 3 as one group. Thus, including rats before MCT injection (W0, control), we had 4 groups' data in the following analysis, as week 1 group (W1), week 2 and week 3 group (W2-3), and week 4 group (W4). We found good separations between groups (Figures 2(b), 2(c), 2(d), and 2(e)), which indicated that metabolic profiles of each separated group are different. The scores plots of OPLS-DA models were performed between W0 and each MCT-injected group (W1, W2-3, and W4), respectively (Figure 3(a), Figure 3(c), and Figure 3(e)). Loading plots of the OPLS-DA models illustrated the significant bins (Figures 3(b), 3(d), and 3(f)). The average Q2 values for the 999 runs were 0.834, 0.786, and 0.820, respectively, for these three OPLS-DA models, suggesting that all these models are well predicted.

We then identified importance of metabolites for class discrimination, as described in Materials and Methods. Metabolites with larger contribution in the discrimination between each stage of PAH and baseline (W0) were selected, respectively, by OPLS-DA loadings and their VIP values. The VIP values and the direction of variation (either increased or decreased) were listed in Table 1, where the distinguished metabolic features were ranked for significance according to their specific VIP values. In the first week, betaine, glycerol, glucose, glycine, and acetoacetate levels increased and creatine, Nac, acetate, choline, and methionine levels decreased. However, in the early stage of PAH (W2-3), glycerol, glucose, betaine, LDL/VLDL, glycine, choline, and acetone levels increased, while lactate, taurine, pyruvate, valine, and acetoacetate levels decreased. In a persistent PAH stage (W4), there were increased serum levels of lactate, creatine, pyruvate, betaine, glycine, choline, glycerol, isoleucine, leucine, and carnitine, while serum levels of LDL/VLDL, glucose, and taurine deceased.

### 3.3. Shifts of Metabolites Profile in Different Stages

**Figure 4:**
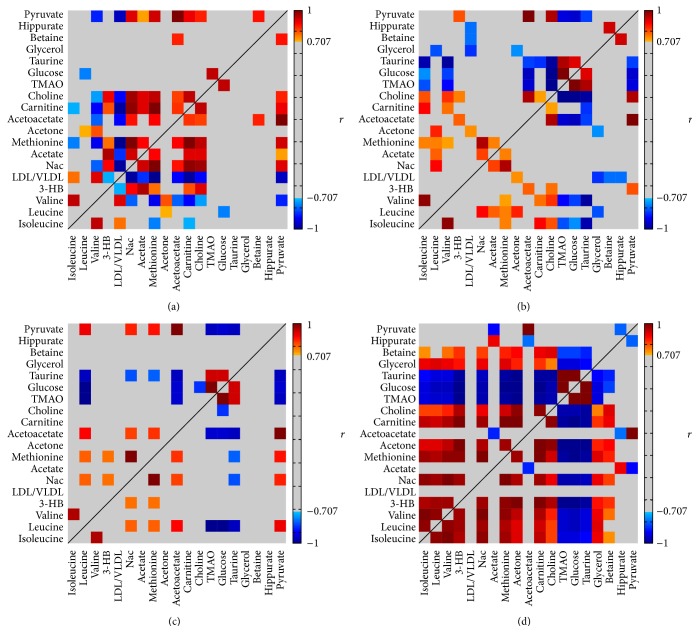
A color heat map of the Pearson's correlation coefficients computed for the characteristic metabolites observed during the PAH progress. The correlation coefficients between the metabolites at each stage: W0 (a), W1 (b), W2-3 (c), and W4 (d). Correlation coefficients were shown with continuous gradient colors, where significant positive correlation is marked in red, negative is in blue, and grey represents no significant correlation being found. Color bars represent the significance of the correlation coefficients.

After the multivariate analysis, we also analyzed statistic differences of the relative concentrations of these selected metabolites among groups, as seen in Table 1. In addition to comparing the data in terms of each single metabolite that changes with respect to the PAH development, we also performed a pairwise correlation to find correlated metabolites and tested the differences of these correlations among different stages of PAH. The differential correlations between these indexes are graphically presented in Figure 4. Briefly, it was found that the correlations shown in W0 had been changed in week 1. In the W2-3, however, some correlations, which were found in the W0, become weak. The profound correlation was found in PAH (W4), in which correlations coefficient become statistically significant between some metabolites, indicating that these metabolites related metabolic pathways might be overactivated in PAH.

### 3.4. Pathway Enrichment Analysis and Dysfunctional Metabolic Pathways Involved

**Figure 5:**
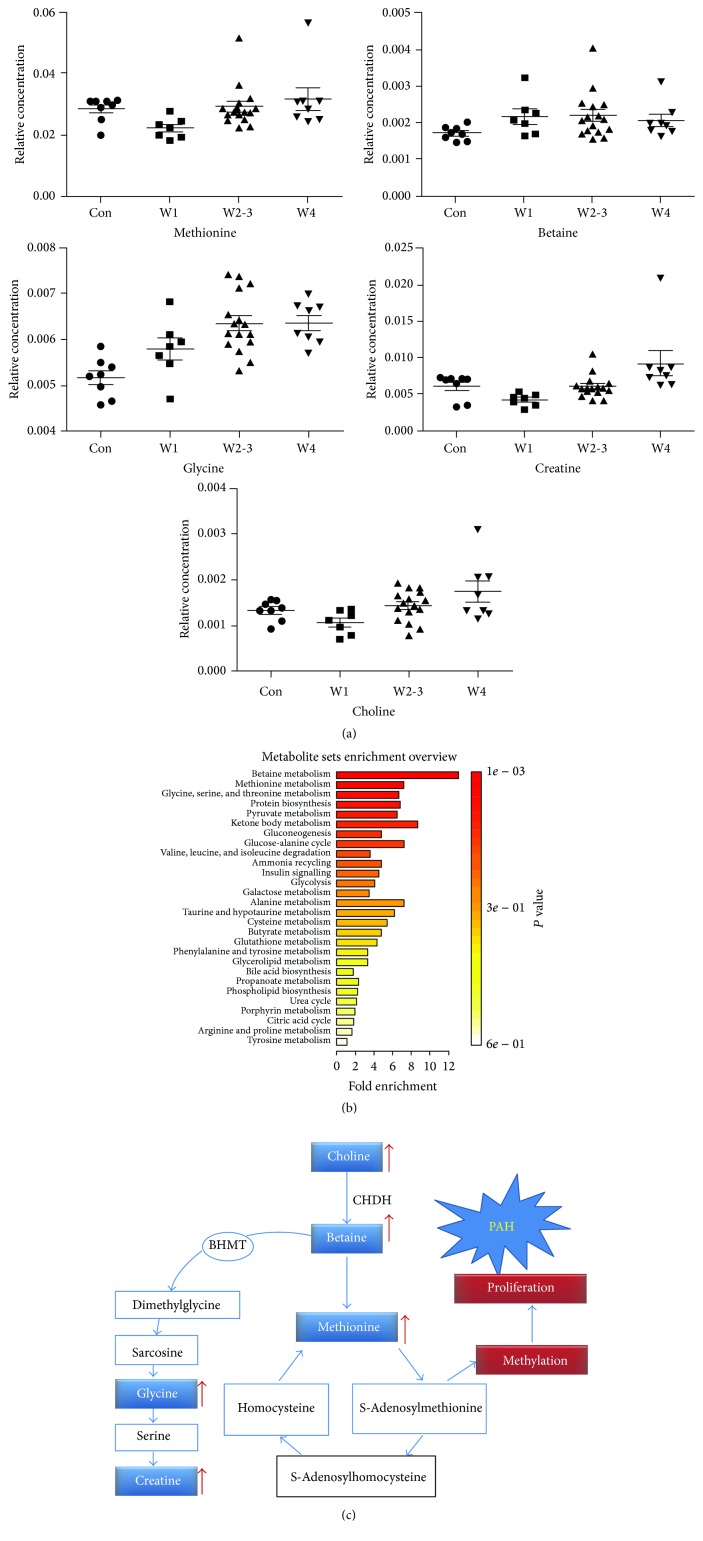
Metabolic abnormality may be a fundamental mechanism of the uncontrolled proliferative and antiapoptotic pulmonary artery smooth muscle cells in the pathogenesis of PAH. (a) Pathway enrichment analysis for determining which pathways are more likely to be involved in the PAH development. (b) Change of metabolite levels during progression of PAH. (c) Hypothetical pathways for choline, betaine, methionine, and energy metabolism dysfunction in PAH. Increased levels of choline and betaine could cause energy metabolism abnormality by affecting mitochondrial function. In PAH, PASMCs proliferation maximizes the usage of methionine for protein synthesis which might reduce the methionine level (choline dehydrogenase: CHDH; methylene tetrahydrofolate dehydrogenase: MTHFD), although methionine is the metabolic production of betaine.

Characteristic metabolites and their concentrations were imported to the web-based tool MetaboAnalyst, to exploit the most disturbed metabolic pathways via Pathway enrichment analysis (Figure 5(a)). Betaine, methionine, and glycine metabolisms were highlighted as vital pathways, which suggested that a disruption of methionine metabolic pathway might contribute to the onset of PAH. Differences of the characteristic metabolites involved in these pathways were given in Figure 5(b). Upregulation of the choline, betaine, and methionine pathways and energy metabolism could be significant pathological mechanism of PAH (Figure 5(c)).

## 4. Discussion

In this study, it was shown that serum metabolite levels varied and closely related to the pathophysiological changes in different stages of PAH. Changes of metabolic profile of glycolysis, lipid metabolism, and methionine were the most significant determinants in different stages of PAH. Correlation between these metabolites and other pathological indexes from different stages of PAH was quite strong, especially in the fourth week, in which glycolysis, lipid, and methionine metabolism pathways were found to be highly activated.

### 4.1. Metabolic Profiling during PAH Progress

Although there were some studies focusing on the metabolic dysfunction in PAH [21–25], just few monitored global and dynamic metabolic shifts in different stages of PAH. Therefore, in this paper, we mainly focused on the changes in serum metabolites during PAH development.

Generally speaking, we found that most of the characteristic metabolites changed significantly in the first week after MCT treatment but tended back to control level in the following weeks. In the first week, the possible explanation for metabolic shifting might be the fact that MCT injection provokes an acute stress response. This could be reflected by reduced level of N-acetyl-L-cysteine, which was believed to play a key role in preventing and suppressing oxidative stress and inhibiting the apoptotic pathways [26, 27].

In the early stage of PAH (weeks 2-3), there was an increase in glucose level; however, there was a decrease in both lactate and pyruvate levels, indicating that both mitochondrial glucose oxidation and glycolytic metabolism were downregulated during this stage.

Furthermore, we found that enhanced lipid metabolism was one of the main biochemical characteristics which may be used to indicate an early onset of PAH. Our data suggested that the disorder of metabolism that occurred in the development of pulmonary hypertension shifted from glucose metabolism to fatty acid usage dominant on the whole. Based on our data, it is believed that a well-known mechanism, Randle cycle, defined as the glucose oxidation switching the energy production from carbohydrates to fatty acid [28], was involved in development of PAH. By this mechanism, glucose oxidation and fatty acid oxidation are balanced. However, to what extent this data may refer to the metabolism occurring in the tissue level remains to be further identified. It seems that our findings that serum glucose level decreased in 2-3 weeks after MCT injection were not consistent with two previous studies [29, 30]. In these two studies, the authors scanned PAH patients and animal models by positron emission tomography (PET); they found that glucose utilization was increased in both the myocardium of PAH patients [29] and pulmonary vasculature [30] of PAH rats, which was in close relation to progression of PAH. Differently, the glucose level in the PAH rats was constant within a specified period of time. Logically, the increase in the glucose in the tissue level might lead indirectly to a decrease in the serum glucose. And this inference may not necessarily be true, because it is also possible that under a specific circumstance both local and circulating level of glucose may be at a higher level at the same time. Also, to what extend the data from animal studies may represent the human pathological process of pulmonary hypertension is still not known at this stage. Because of the discrepancies between rats and humans in relation to glutathione homeostasis, for example, higher primates including humans cannot synthesize ascorbate, while rats can [31]. As a result, implications on the translatability of the presented results should be with caution.

In agreement with previous works [32], we found an upregulated glycolysis and downregulated glucose oxidation in the sustaining PAH stages (W4). Glucose level reduced in PAH, suggesting an excess consumption of glucose as energy substrates, which leads to the deficiency of glucose in peripheral blood. Inherent to the endothelial proliferation in PAH, the global metabolic shift favors the production of ATP via glycolysis at the expense of oxidative phosphorylation in response to hypoxia even with adequate oxygen [21, 33], known as Warburg Effect, which was firstly described in tumor cells. The hypoxia-inducible factor (HIF) masters the hypoxic response and has long been known to play an important role in the PAH development, via overactivation of glycolytic enzymes and suppression of glucose oxidation [33, 34].

### 4.2. Choline, Betaine, and Methionine Metabolism

Interestingly, we also found an increase in the level of choline, betaine, methionine, and glycine. Furthermore, betaine and methionine pathway was identified as vital pathway in PAH. Importantly, imbalance of metabolism in choline, betaine, and methionine is involved in the pathways of cells proliferation and energy metabolism [35–37]. Similar to cancer cells, activation of cell proliferation is one of the major pathophysiological mechanisms of PAH. In 1998, Voelkel and colleagues firstly reported a cancer model for primary pulmonary hypertension by showing active overgrowing, vasculogenesis, and angiogenesis in endothelial cells of PAH [38]. Accumulating evidences demonstrated that the vascular remodeling in PAH rats is the consequence of cell proliferation and antiapoptosis of the smooth muscle cells in pulmonary artery, to some extent, similar to tumor cells [39, 40].

Although the statistical correlations and the pathway enrichment analysis did not provide cause-effect relationship directly, accumulating data suggested a possible underlying mechanism. Choline is known as a precursor for formation of the neurotransmitter acetylcholine which can be oxidized to form betaine (catalyzed by choline dehydrogenase, CHDH). It is well-known that betaine is a methyl donor in the formation of methionine, a critical step in the formation of methyltetrahydrofolate catalyzed by methylene tetrahydrofolate dehydrogenase (MTHFD) [36]. Methionine is vital for protein synthesis in PASMCs proliferation of PAH, similar to phenomenon in hepatocarcinogenesis [35]. Additionally, high level of choline and betaine could also lead to abnormal mitochondrial structural and functional changes, resulting in disorders of energy metabolism (Figure 5).

Given that PAH is a fetal disease with very poor survival prognosis, the research of its specific metabolic mechanism may shed light on potential therapeutic strategies for PAH [5, 33]. Intervention of the metabolic dysfunction may help to inhibit the uncontrolled cell proliferation in PAH. For example, abolished fatty acid oxidation by deleting the gene that encodes the regulating enzyme malonyl-coenzyme A decarboxylase (MCD) may shift the metabolic imbalance back to glucose oxidation; as a result, the mice did not develop pulmonary hypertension after MCT treatment or induced hypoxia condition [41]. However, to what extent the metabolites we selected and their relevant metabolic pathways are linked to factors such as the uncontrolled proliferative and antiapoptotic pulmonary artery smooth muscle cells in the pathogenesis of PAH still remains to be established.

## 5. Conclusion

At this stage, we used ^1^H NMR-based metabonomics to scan metabolic shifts in the sera of PAH rats during progression of vascular remodeling. The preliminary results provided valuable knowledge on the biochemistry during PAH process and highlighted the betaine and methionine pathway in the onset of PAH, which could be helpful for further research of PAH pathogenetic mechanism and treatment.

## Figures and Tables

**Table 1:** Changes of metabolites after MCT injection.

Chemical shift (ppm)	Metabolites	W1 versus W0				W2-3 versus W0				W4 versus W0			
		|*r*|^a^	VIP^b^	Vary^c^	*P* ^d^	|*r*|^a^	VIP^b^	Vary^c^	*P* ^d^	|*r*|^a^	VIP^b^	Vary^c^	*P* ^d^
3.70	Glucose	0.72	3.21	↑↑		0.52	4.04	↑		0.58	3.03	↓	
1.34	Lactate	—	—	—		0.58	4.23	↓↓		0.73	8.77	↑↑	0.008
2.37	Pyruvate	0.69	1.1	↑↑		0.62	2.15	↓↓		0.79	4.15	↑↑	0.050
0.92	LDL/VLDL	—	—	—		0.64	2.4	↑↑		0.58	1.77	↓	
3.67	Glycerol	0.85	3.27	↑↑	<0.001	0.89	4.18	↑↑	0.016	0.59	2.22	↑↑	0.001
1.20	3-Hydroxybutyrate (3-HB)	0.8	1.18	↓↓		—	—	—		—	—	—	
2.23	Acetone	0.76	1.26	↑↑		0.56	1.26	↑↑	0.039	—	—	—	
2.29	Acetoacetate	0.82	1.74	↑↑		0.62	1.74	↓↓		—	—	—	0.042
1.92	Acetate	0.86	2.59	↑↑	0.004	—	—	—		—	—	—	
2.45	Carnitine	0.84	1.24	↓↓		—	—	—		0.52	1.33	↑↑	
3.23	Taurine	—	—	—		0.48	3.11	↓		0.56	2.48	↓	0.025
2.14	Methionine	0.77	1.96	↓↓		—	—	—		—	—	—	
3.20	Choline	0.81	2	↓↓		0.57	1.68	↑↑		0.69	2.29	↑↑	
3.89	Betaine	0.8	4.95	↑↑		0.51	2.76	↑	0.039	0.54	3.8	↑	
3.56	Glycine	0.64	2.79	↑↑	0.043	0.54	1.87	↑↑	<0.001	0.62	3.19	↑	<0.001
3.04	Creatine	0.66	2.63	↓↓		—	—	—		0.57	4.48	↑	0.018
0.99	Leucine	—	—	—		—	—	—		0.54	1.44	↑	
1.02	Isoleucine	—	—	—		—	—	—		0.68	1.86	↑↑	0.044
1.04	Valine	0.69	1.65	↑↑		0.47	2.01	↓		—	—	—	
2.04	N-Acetyl-l-cysteine (Nac)	0.69	2.31	↓↓		—	—	—		—	—	—	

^a^Metabolites shift correspond to signals clearly distinguished in the loadings profile (not necessarily representing complete spin systems); ^b^VIP list produced by OPLS-DA; ^c^Increased or decreased when comparing to control (W0), ↓ or ↑, *p* < 0.05, ↓↓ or ↑↑ *p* < 0.01; ^d^ *P* values, when comparing to control (W0).
